# Resveratrol food supplements: a survey on the role of individual consumer characteristics in predicting the attitudes and adoption intentions of US American and Danish respondents

**DOI:** 10.1186/s12889-015-1348-7

**Published:** 2015-02-10

**Authors:** Jessica Aschemann-Witzel, Klaus G Grunert

**Affiliations:** MAPP Centre for Research on Customer Relations in the Food Sector, Aarhus University, Bartholins Allé 10 building 1323-321, 8000 Aarhus, Denmark

**Keywords:** Consumer, Resveratrol, Supplement, Health, Individual, Interest, Attitude, Intention, Behaviour, Alternative medicine

## Abstract

**Background:**

Consumers increasingly choose food supplements in addition to their diet. Research on supplement users finds they are likely to be female, older and well-educated; Furthermore, supplement users are often characterised as being especially health-oriented, an observation which is termed the ‘inverse supplement hypothesis’. However, results are dependent on the substance in question. Little is known so far about botanicals in general, and more specifically, little is known about resveratrol. The psychographic variables of food supplement users are yet relatively underexplored. By comparing US and Danish respondents, we aimed to identify whether sociodemographic variables, health status, health beliefs and behaviour and interest in food aspects specifically relevant to resveratrol (e.g., naturalness, indulgence, and Mediterranean food) explain favourable attitudes and adoption intentions toward resveratrol supplements.

**Methods:**

A survey sent to a representative online panel in the United States and Denmark was analysed using linear regression.

**Results:**

We find that sociodemographic variables contribute little to explaining favourable attitudes toward and adoption intentions of resveratrol supplements, except for the negative association with higher education in the United States. The inverse supplement hypothesis was not confirmed. Belief in the favourable health effects of resveratrol and usage of complementary and alternative medicine positively affect attitudes and adoption intention. An interest in the indulgence dimension of food explains positive attitudes in the United States and adoption intentions in both countries.

**Conclusions:**

The results indicate that potential consumers of resveratrol supplements are identified by their usage of complementary and alternative medicine, rather than by sociodemographic variables. They are not characterised by especially healthy behaviours, which contradicts the inverse supplement hypothesis. Instead, potential consumers of resveratrol supplements may be characterised by their focus on the indulgence dimension of food.

**Electronic supplementary material:**

The online version of this article (doi:10.1186/s12889-015-1348-7) contains supplementary material, which is available to authorized users.

## Background

### Introduction and research questions

Nowadays, citizens in industrialized countries can look forward to a high life expectancy. The crucial element for being able to enjoy this long life is good health. Public-policy makers aim to improve citizens’ health to ensure quality of life during aging, if not to reduce costs in the healthcare system. Much of the responsibility for a person’s health status rests, though, on the individual: day-to-day decisions about what to eat, in which amount and combination and in conjunction with how much physical activity yield differing degrees of healthy lifestyles. People are increasingly willing to take control of their diets [1,2]. Available options include products with enhanced health properties, such as functional food [3-5] and food supplements [6].

Supplement users fit a certain sociodemographic profile, e.g. tend to be females, well-educated, and older [7]. In addition, it has been observed that supplement users appear to lead a relatively healthy lifestyle [8]. Thus, they do not necessarily constitute the segment in greatest need of supplements, an observation which is termed the ‘inverse supplement hypothesis’ [9]. The probable cause of this is that consumers motivated and able to lead a healthy lifestyle are more inclined to and capable of adopting supplement usage behaviour. Supplement usage might be related to the increasing popularity of complementary and alternative medicine (CAM), which is driven by the search for a more self-determined and holistic approach to health, well-being and lifestyle [10,11]. However, it has been observed that consumers equate the ‘naturalness’ of e.g. botanical supplements with ‘safety’ [2], and therefore, at times do not inform their doctors about their supplement use, a fact that has caused concern about adverse health effects [2,12]. A range of factors may influence how consumers view supplements, e.g., the type of product or substance [13], the perceived relevance of the effect [13], beliefs and expectations about health outcomes [9]. More general factors might also have an impact on supplement use, such as the prevalence of use in the respective country [7], or for example, the degree of celebrity use and endorsement [14]. Supplement use or the type of supplement chosen might furthermore be influenced by individual attitudes and interests within the food and health domain, such as the degree of preference for naturalness [15] or interests in certain food types and dietary trends [3,16]. As the influence of the factors listed above might vary considerably, depending on the substance and the individual in question [13], there is a need to test the interrelation for specific supplement groups or substances. In addition, psychographic variables have not yet been explored thoroughly in the analysis of food supplement users [7].

The aim of this paper is to analyse the role of individual characteristics in determining favourable attitudes and adoption intentions toward the resveratrol supplement in two countries, namely the United States and Denmark. Resveratrol is as yet relatively unknown in Denmark, while it is a rather established substance among the food supplement range in the United States (see [17]), which is why the between-country comparison can shed light on the differences between a developed and an emerging food supplement market. Resveratrol is a substance that may help reduce the risk of chronic diseases such as osteoporosis, cancer and Alzheimer’s disease; it is also said to potentially alleviate the negative effects of obesity, aging and lack of exercise [18-21]. It is a substance naturally found in grapes and wine and has been discussed in relation to the so-called ‘French paradox’ [22,23]. The French paradox is the observation that even though there is a high intake of saturated fats in France, the mortality rate from coronary heart disease is low. This effect was later linked to the traditional diet of the Mediterranean region (i.e., rich in whole-grain cereals, fruits, vegetables, wine, fish, legumes, nuts, and olive oil, and moderate in meat, see [24]) and might be one of the reasons for the popularity of Mediterranean cuisine. Because of its varying possible effects and associations, resveratrol is a substance especially interesting to study, with regard to how psychographic constructs are related to food-impact health behaviours. In a departure from previous research, the following four research questions are addressed:

Are US and Danish consumers who have favourable attitudes and adoption intentions toward a botanical food supplement containing resveratrol…

1. … characterised by the same sociodemographic variables as has been found for other supplements or supplements in general (e.g., being female, relatively older, and well-educated)?
2. … of good health status and behaviour, indicating that the ‘inverse supplement thesis’ applies to the supplement in question?
3. … expressing favourable health beliefs and behaviours, indicating that they believe in non-medicinal actions (expectations about the health effects of resveratrol, holistic health beliefs, and reporting the use of CAM)?
4. … characterised by psychographic constructs that can be assumed to explain general favourable reactions to botanicals and more specifically, reactions to resveratrol (e.g., higher interest in natural food, the indulgence dimension of food, and Mediterranean cuisine)?

In the following, we outline the current state of research on the characteristics of supplement users, in relation to each of our four research questions.

### Country differences in usage and sociodemographic characteristics of food supplement users

The use of dietary supplements in industrialised countries is said to have increased over the past decades [8,14,25]. In the United States in the 1990s, around 40% of the adult population was found to be regular supplement users, whereas after the turn of the millennium, as many as 73% claimed to have taken supplements during the previous 12 months [25]. Other studies, however, cite statistics such as 40–48% [12] or 49% (44% male, 53% female [7]) for the United States during the same time frame. The differences in numbers probably stem from differences in the definition of supplements or the definition of regular supplement use. Findings for Europe, though, generally tend to be lower: in 2001, a national survey in the UK stated that 17% of females use supplements [9], a representative survey in the Netherlands established that 30% of the respondents claimed to use functional food or a supplement at least once a week [13], while in Germany, 18% of men and 25% of females used supplements regularly [8]. In Switzerland, however, as much as 41.5% of the population appeared to use supplements [26]. Egan et al. conclude that within Europe, usage is highest in Scandinavian countries and lowest in the Mediterranean, citing results of the EPIC study that found the average share of users ranges from 2–52% among men and from 7–66% among women [7].

With regard to the sociodemographic profile of supplement users, it is rather established that females are overrepresented among supplement users [8,9,25-28]. In addition, results tend to show that higher age [9], higher education [26] and higher income characterise supplement users [9], as Egan et al. also concluded in their review [7]. However, a study focusing on weight loss supplements in the United States found that users were typically only 25–34 years old and of lower education level [27], thus strengthening de Jong et al.’s conclusion that characteristics of users depend on the substance in question [13]. Interestingly, a US study indicated that herbs and botanicals are chosen by men in particular [25], which shows user characteristics might be quite contrary to general findings when focusing on specific supplement types.

### Health status and health behaviour of supplement users

Quite a number of authors comment that supplement users tend to exhibit a relatively healthy lifestyle—termed the ‘inverse supplement hypothesis’ - [2,9,13]. De Jong et al. and Beitz et al. found that supplement users indeed showed a healthier eating pattern for specific indicators in the Netherlands and Germany, concluding however that it depends on the substance [13] and that the healthier eating pattern is not related to a difference in energy intake, but rather, to a difference in food composition [8]. Van der Horst et al. found evidence for the inverse supplement thesis in Switzerland, but at the same time also found support for the opposing hypothesis, because some supplement users were consuming a significantly less healthy diet [26]. In addition, they observed supplement users being relatively less health conscious [26]. This suggests that, apart from user characteristics depending on the supplement substance in question, user groups of the same product might differ in the role that they attach to supplements—as a ‘remedy’ against their existing unhealthy eating patterns or as one of many features of their healthy eating pattern.

### Health beliefs and potential psychographic characteristics of supplement users

It is interesting to note that users of complementary and alternative medicine (CAM) are similarly characterised as food supplement users and tend to be female, well-educated, and relatively older [11]. Considering the similarity of the role of CAM and of supplements—both supporting health in a ‘soft’ way and acknowledging the close ties between nutrition, well-being and health - users of CAM and supplement users can be expected to overlap in their psychographic profile to some extent. It has been found that users of CAM hold strong beliefs about the influence of psychological factors (e.g. ‘state of mind’, stress, well-being [10]) on health. Only a few studies extended research on supplement users to a range of psychological measures and influence factors [7]. An example is an Australian study by Conner et al., who positively tested the explanatory value of the theory of planned behaviour [9]. It has been found that CAM users exhibit modern health worries [10], including worries about genetic modification and pesticides. Modern health worries, in turn, were found to be related to interest in the naturalness of food [29,30], and consumers tend to prefer ‘naturalness’ in foods more than in medicine [15]. Food supplements and botanicals are perceived as playing a role between both of these, and the role of consumer preference, for naturalness in supplements specifically, is not well known. Furthermore, the indulgence aspect of food might play a role in psychological ‘well-being’, which, in the holistic view of food and health relations, is supposed to impact health.

### Research on resveratrol and on botanicals in general

Little research can be found on consumer behaviour in relation to resveratrol, whether as a substance in a supplement or in a food product. To our knowledge, the only study published is one by Barreiro-Hurlé et al. The authors analysed Spanish wine consumers’ preferences for resveratrol-enriched wine with the help of a survey and choice test, and identified a willingness to pay a 55% premium for the health-enhancing wine. However, the respondents regarded wine as generally healthy, although few were able to explain why exactly they thought this to be the case [31]. Interestingly, the health benefits of wine appear to be an emerging research area and point of consumer interest [32,33].

A large share of the supplements used consists of vitamins and minerals [25]. An important and growing area, though, are so-called botanicals [7]. Among the different categories of food supplements, resveratrol-containing products are likely to be subsumed under botanicals. Such plant-based supplements have been examined within the scope of an EU research project [7,34]. It is argued that plant-based supplements are more likely to be used by consumers as a means of medication than as a method to improve nutrition, although it is difficult to disentangle the use for one from the other. From a public-health perspective, this fact calls for more intense scrutiny of consumer behaviour and the characteristics of users of this kind of supplement [7].

## Methods

### Survey method and measures

An online survey was administered in the United States and Denmark. Only adult respondents were included. An introductory text explained the aim, the anonymous, confidential and non-commercial use of the study for research purposes only and what the survey consisted of, and provided a contact person for any further questions. Proceeding to fill out the online survey after this step was assumed to equal written consent; a withdrawal from the survey was possible at all stages during the survey. The research followed the ethical principles as outlined by the Helsinki declaration and further explained in the research group’s ethical guidelines^a^.

Respondents read a description of the resveratrol food supplement and responded with their attitudes and adoption intentions. Their individual characteristics were measured in the following way. First, to identify sociodemographic characteristics (research question 1), we collected the respondent’s country of residence, gender and age in years. Respondents were asked to state their level of education, which was classified into a binary variable indicating whether they had achieved a higher (university) education or not. Then, to identify the respondent’s actual and self-reported health status and their health behaviour, and thereby explore the inverse supplement hypothesis (research question 2), they were asked to reveal their height and weight for calculating their body mass index (BMI). Respondents further assessed their fitness and their health status, which was merged into one self-reported indicator of ‘fitness and health’. For health behaviour, we used an indicator for ‘healthy lifestyle practices’ consisting of a 14-item measure [35]. It was chosen for its brevity and for encompassing various aspects of health behaviour. Additionally, the measure had been developed in the United States, where a part of the survey was to take place.

Next, to assess the respondent’s health beliefs and respective behaviour indicating their belief in non-medicinal actions in favour of health (research question 3), the following measures were used: to assess respondents’ belief in the health effects of the supplement, they rated 10 statements describing the potential health effects of the product with which they were presented. The statements were derived from the literature [18-21]. Furthermore, respondents rated their beliefs about health needing to be approached ‘holistically’ — understood as the relevance of psychological factors for physical health — using four statements selected from Furnham, on attitudes toward science and medicine, and Hyland et al., on holistic health (choosing the two statements loading highest in each case) [10,36]. Finally, respondents were asked to state the frequency with which they used complementary and alternative medicine during the past year (assessed on a seven-point Likert scale ranging from ‘never’ to ‘regularly’).

In addition, we measured psychographic constructs on interest in food characteristics that are specifically relevant to the substance resveratrol (research question 4). We used the established ‘natural product interest’ measure of Roininen et al., which is a sub-dimension of an attitude scale on health and hedonic characteristics of food, developed through qualitative and representative quantitative surveys [37], and has been tested repeatedly since its introduction [38]. In addition, we selected three statements from the ‘health discourse’ measure developed by Chrysochou et al., on the basis of an ethnographic study by Kristensen et al. [39,40]. These constitute the sub-dimension of ‘indulgence’, and were used as a measure of interest in the indulgence aspect of food. Furthermore, we asked respondents to assess their liking of the Mediterranean cuisine as one among other cuisine examples (Mediterranean-inspired, Danish or American respectively, Asian, other).

As dependent variables, we measured ‘attitude toward the product’ with three established statements taken from previous literature [41,38]. We also asked respondents to assess their likelihood of adopting intake of the product within the coming year, as a measure of their intentions to use.

### Data collection and analysis

The online survey was conducted in December 2012. The link to the survey was sent to respondents of a panel from a market research company, applying a quota for age, gender and region. The panel is representative of the Danish and US populations. Respondents were screened for supplement use to exclude individuals who were non-users or who had too little experience with food supplements. Respondents stating that they had ‘never’ taken, or taken supplements ‘a few times’ in the last year, were excluded. Before beginning the survey, an introductory page briefly introduced resveratrol as follows:

“With this survey, we would like to explore your opinion about dietary supplements containing resveratrol, and what their role could be in your daily food and nutrition habits. Resveratrol can be found in various foods but especially in red grapes, and therefore also in red wine, which is often the reason somebody might have heard of it. It appears to have a number of potential benefits for health. Resveratrol is discussed in relation to diseases, such as certain cancer types, cardiac disease, dementia including Alzheimer’s disease, diabetes, obesity and general aging.”

We did not test for the respondents’ comprehension of resveratrol and its effects.

The survey was originally completed by 1048 respondents, 527 in Denmark and 521 in the United States, with a mean completion duration of 16.3 min in Denmark and 13.9 min in the United States. We assumed that spending less than 5 min signified respondents did not read the product descriptions sufficiently or deliberate over the questions; therefore, we excluded the 4% of respondents from the dataset who spent less than 5 minutes on the survey. Overall, 11% actually had used resveratrol supplements previously (3% in Denmark, 18% in the United States). We decided to exclude these respondents to achieve a more homogenous sample of supplement users who share a lack of experience with the exact substance presented. The analyses below were calculated using a sample size of 454 in Denmark and 376 in the United States (see Table 1). The two countries were analysed separately. We conducted linear regression to test for the relationships of all independent variables (enter procedure, see Table 2) with (1) the dependent variable of attitude and (2) the dependent variable of adoption intention (see Table 3). We used SPSS 21 for the analysis. All independent variables were checked for multi-collinearity, and VIF-values were below |2|.

**Table 1:** **Sample characteristics for each variable and per country**

**Variable**	**Denmark (454)**	**US (376)**	**Sample comparison**
Gender, female	61.0%	51.1%	χ^2^(1) = 8.284, *p* = .004
Age, years	45.7	42.0	*t*(828) = 3.982, *p* = .000
Education, high	45.2%	51.6%	χ^2^(1) = 3.419, *p* = .064
BMI, *M*	25.7	27.4	*t*(762) = −3.658, *p* = .000
Self-reported fitness and health, *M* (*SD*) (2 items)	4.40 (1.30)	4.60 (1.20)	*t*(828) = −2.599, *p* = .010
Health behaviour, *M* (*SD*) (14 items)	4.30 (0.56)	4.23 (0.67)	*t*(804) = 1.668, *p* = .096
Health expectation, *M* (*SD*) (10 items)	3.68 (1.06)	4.38 (1.07)	*t*(828) = −9.364, *p* = .000
Holistic health belief, *M* (*SD*) (4 items)	5.29 (0.81)	5.43 (0.92)	*t*(828) = −2.283, *p* = .023
CAM use, *M* (*SD*)	2.50 (1.50)	2.10 (1.20)	*t*(828) = 4.390, *p* = .000
Natural product interest, *M* (*SD*) (6 items)	4.29 (1.20)	4.17 (1.11)	*t*(828) = 1.476, *p* = .140
Food indulgence interest, *M* (*SD*) (3 items)	4.50 (1.34)	4.51 (1.34)	*t*(828) = −.019, *p* = .985
Mediterranean cuisine interest, *M* (*SD*)	5.37 (1.59)	4.83 (1.72)	*t*(828) = 4.633, *p* = .000
Attitude, *M* (*SD*) (3 items)	4.17 (1.21)	4.99 (1.17)	*t*(828) = −9.752, *p* = .000
Adoption intention, *M* (*SD*)	2.72 (1.75)	3.83 (1.78)	*t*(828) = −9.034, *p* = .000

*Notes. n* = 830. BMI could only be calculated for respondents who opted to provide their weight and height.

**Table 2:** **Independent measures**

**Measure**	**Items**	**Cronbach’s alpha US - DK**	
Self-reported fitness and health	How do you assess your own fitness?	0.748	0.790
	How do you assess your own health status?		
Health behaviour	14 items from [34]^1^	n/a	n/a
Health expectation	10 items derived from the literature^1^	0.916	0.915
Holistic health belief	People sometimes feel worse rather than better after orthodox/conventional medical treatments.	0.728	0.607
	State of mind is a crucial part of achieving better health—positive thinking can enhance physical health.		
	The symptoms of an illness can be made worse by depression.		
	When people are stressed, it is important that they are careful about other aspects of their lifestyle, as their body already has enough to cope with.		
CAM use	How often have you used what you would consider alternative or complementary medicine (medicine and/or treatments) during the last year?	-	-
Natural product interest	6 items from [36]^1^	0.774	0.787
Food indulgence interest	I am inspired in my cooking by foreign cuisine and other new ideas.	0.828	0.765
	I am willing to spend extra time on my daily food consumption for the sake of culinary pleasure.		
	I am willing to spend extra money on my daily food consumption for the sake of culinary pleasure.		
Mediterranean cuisine interest	To what extent do you like the following cuisines/cooking styles? [Mediterranean-inspired]	-	-

*Notes*. All statements were measured on a seven-point scale, except for statements 1–11 from the health behaviour measure, which were measured on a 1 (*never*) to 9 (*more than seven times*) scale. The statements used for measuring health expectations can be provided upon request. Cronbach’s alpha was not computed for the health behaviour measure, as we consider this a formative indicator. ^1^Please see Additional file 1: Table S1 for these statements.

**Table 3:** **Dependent measures**

**Measure**	**Items**	**Cronbach’s alpha US - DK**	
Attitude	Taking this product is …	0.908	0.905
	1 = extremely bad, 7 = extremely good		
	I am [strongly against/strongly for] taking this product.		
	1 = strongly against,7 = strongly for		
	I [dislike/like] taking this product.		
	1 = dislike, 7 = like		
Adoption intention	How likely is it that you will take resveratrol supplements during the coming year?	-	-

*Notes*. All statements were measured on a seven-point scale.

## Results and discussion

Averages for the measures for the two countries are displayed separately in Table 1. US respondents showed significantly more favourable attitudes and favourable adoption intentions. Furthermore, they self-reported better general fitness and health, while Danish respondents tended to achieve a higher health behaviour score. US respondents had considerably higher health expectations for resveratrol supplements and expressed stronger holistic health beliefs. Danish respondents, in turn, reported more CAM use and expressed a higher interest in Mediterranean cuisine. Respondents from the two countries did not differ in the natural and food indulgence interest. Below, the results of the regression (see Table 4 for the results on attitude and Table 5 for the results on adoption intention) are presented for each of the four research questions.

**Table 4:** **Influence of individual characteristics on attitudes toward a resveratrol supplement, linear regression**

**Independent variables**	***B***	***t***	***p***	***B***	***t***	***p***
	**DK:**			**US:**		
	**R** ^**2**^ **= .401 (adjusted .383);**			**R** ^**2**^ **= .409 (adjusted .387);**		
	**F (12) = 22.118, p = .000**			**F (12) = 18.420, p = .000**		
Gender	.116	1.110	.268	-.047	-.450	.653
Age	-.004	−1.112	.267	.005	1.329	.185
Education	.118	1.191	.234	-.328	−3.061	.002
	R^2^ = .004 (−.003), F(3) = .531			R^2^ = .025 (.017), F(3) = 3.199		
BMI	.001	.067	.947	.013	1.525	.128
Reported fitness & health	-.001	-.027	.979	.048	.912	.363
Health behaviour	-.013	−1.809	.071	-.010	−1.587	.114
	R^2^ = .015 (.007), F(3) = 2.011			R^2^ = .004 (−.005), F(3) = .401		
Health expectation	.683	14.479	.000	.567	11.065	.000
Holistic health beliefs	.065	1.033	.302	.113	1.781	.076
CAM use	.087	2.649	.008	.125	2.837	.005
	R^2^ = .364 (.360), F(3) = 85.788			R^2^ = .350 (.345), F(3) = 66.746		
Natural product interest	-.003	-.061	.951	-.090	−1.685	.093
Food indulgence interest	.002	.040	.968	.139	3.131	.002
Mediterranean cuisine	.065	1.845	.066	.032	.979	.328
	R^2^ = .022 (.016), F(3) = 3.404			R^2^ = .062 (.054), F(3) = 8.132		

*Note*: R^2^ and F values are given for each block of variables.

**Table 5:** **Influence of individual characteristics on intention to adopt a resveratrol supplement, linear regression**

**Independent variables**	***B***	***t***	***p***	***B***	***t***	***p***
	**DK:**			**US:**		
	**R** ^**2**^ **= .322 (adjusted .301);**			**R** ^**2**^ **= .411 (adjusted .389);**		
	**F (12) = 15.685, p = .000**			**F (12) = 18.539, p = .000**		
Gender	.235	1.489	.137	.070	.436	.663
Age	.009	1.493	.136	.005	.767	.444
Education	.216	1.446	.149	-.360	−2.194	.029
	R^2^ = .008 (.002), F(3) = 1.280			R^2^ = .012 (.004), F(3) = 1.447		
BMI	.013	.861	.390	.008	.637	.525
Reported fitness & health	-.083	−1.188	.236	-.121	−1.508	.132
Health behaviour	-.017	−1.468	.143	.007	.742	.459
	R^2^ = .025 (.018), F(3) = 3.437			R^2^ = .018 (.009), F(3) = 2.003		
Health expectation	.792	11.091	.000	.828	10.567	.000
Holistic health beliefs	.057	.603	.547	-.130	−1.333	.183
CAM use	.201	4.022	.000	.266	3.966	.000
	R^2^ = .283 (.278), F(3) = 59.150			R^2^ = .323 (.317), F(3) = 59.105		
Natural product interest	-.052	-.755	.451	-.025	-.311	.756
Food indulgence interest	.127	2.044	.042	.348	5.135	.000
Mediterranean cuisine	.009	.165	.869	-.067	−1.312	.191
	R^2^ = .018 (.012), F(3) = 2.796			R^2^ = .104 (.096), F(3) = 14.332		

*Note*: R^2^ and F values are given for each block of variables.

### Results

With regard to research question 1, i.e., the influence of sociodemographic characteristics on attitudes toward the product and adoption intent, the results show that gender, age and education were not explanatory factors, except for a small effect of education in the United States—higher education had a significant negative influence on attitudes (Table 4) and adoption intention (Table 5). For research question 2, where we tested indicators for the inverse supplement hypothesis, BMI, self-reported fitness and health status and the health behaviour indicators were not related to resveratrol attitudes and adoption intention. In Danish respondents, having favourable health behaviours was linked negatively to attitudes toward the product, but this was only marginally significant at *p* < .10 (Table 4).

Looking at the variables for research question 3 (e.g., health effect beliefs, CAM use, related holistic health belief), the results show that favourable expectations about the health effects of the product were significantly and positively correlated with attitude and adoption intention in both countries. Current usage of complementary and alternative medicine was also highly associated with favourable attitudes and intentions in both Denmark and the United States (Table 4 and 5). In the United States, respondents agreeing to statements describing a holistic health belief were more likely to express favourable attitudes toward the resveratrol food supplement, but this was only marginally significant at *p* < .10 (Table 4).

Finally, the variables included under research question 4, which aimed to explore psychographic measures potentially relevant to the perception of resveratrol supplements, were considered. We did not find a significant link to natural product interest, apart from observing a tentative negative association with attitudes in the United States (*p* < .10). Reporting an interest in the indulgence aspect of food, however, was significantly and positively related to both attitudes and adoption intentions (Table 4 and 5). In Denmark, however, a preference for Mediterranean cuisine tended to be related to more favourable attitudes, but only marginally significant at *p* < .10.

### Discussion

US respondents’ far more positive reactions toward resveratrol, in both their attitudes and adoption intention, matched our expectations, given that the prevalence of supplement use overall, and of resveratrol use in particular, is much greater in the United States than in Denmark [7]. Furthermore, there were far greater health expectations of the product among US respondents, which was linked to their attitudes and intentions. The factors linked to favourable attitudes and adoption intentions are, however, very similar between the two countries, except for the negative association with higher education that was found only in the United States. Therefore, it appears as if resveratrol supplement usage is largely correlated to similar factors in the two countries.

Sociodemographics were not significantly linked. This is in contrast to the literature, which suggests that supplement users tend to be well-educated, female and relatively older [7]. Neither did we find men to be more favourable toward resveratrol, contrary to Timbo et al., who indicated that men might be more likely to prefer botanicals [25]. However, the sample consisted of (at minimum) occasional users of supplements, so the results need to be interpreted as factors linked to the perceptions of resveratrol supplements among supplement users, and not in the population at large. It is surprising, however, that we found a small but statistically significant negative association with education in the United States, as this is counter to previous research on supplement users’ characteristics.

The inverse supplement hypothesis suggests that consumers who are in the least need of supplements are more likely to use supplements to boost their already favourable health state or behaviour [2,9,13]. However, our findings do not support this hypothesis, at least not with the indicators that were used. Thus, the inverse supplement hypothesis either could not be uncovered by the approach taken, or does not apply to the botanical resveratrol. It is unknown whether the inverse supplement hypothesis might be more applicable to one type of supplement more than others, i.e., depending on whether it is regarded as being closer to medicine—as botanicals are—or closer to food.

The most strongly associated factor appears to be high expectations of the effects of the supplement on health, which we expected. However, it was also found that respondents who reported using complementary and alternative medicine were most likely to be favourable in both attitudes toward and adoption intentions for the resveratrol supplement. We have not found this relation shown in the literature so far, apart from the fact that the sociodemographic characteristics of both CAM and supplement users are rather similar [11]. The finding might indicate that the same respondents are leaning toward the usage of supplements, as well as their choice of complementary and alternative medicine, or even perceive the former belonging to the latter.

No positive relations were found between natural product interest and resveratrol food supplement interest; thus, we cannot confirm that a preference for ‘naturalness’ [15] is linked to interest in resveratrol supplements as a plant-based supplement. Interestingly, however, we found that indulgence food interest was associated positively with attitude and adoption intention. Thus, the results show that it is possible to identify psychographic variables of relevance. Resveratrol is associated with red wine and the related Mediterranean diet, as well as with alleviating the negative effects of obesity. Both are, in turn, associated with the enjoyment of food, especially when thinking about the role of food in France [42] and the French paradox [22,23], and given that many consumers apply the taste = unhealthy ‘intuition’ [43]. Our results suggest that the respondents who were interested in food enjoyment were, in fact, also more interested in the resveratrol food supplement. Interestingly, this indirectly indicates a contradiction of the inverse supplement hypothesis, assuming the foods these consumers were envisioning while answering the statements were relatively unhealthy.

A limitation of the study is that the cross-sectional nature of the data does not allow for understanding developments over time with regard to this issue. While the panel was representative, our screening for supplement use, and the quota sampling of respondents, mean that the sample was not representative of the US or Danish population.

## Conclusions

We conclude that among consumers who at least occasionally use supplements, strong belief in the products’ health effects, CAM usage behaviour and interest in the indulgence dimension of food are most strongly linked to favourable attitudes and adoption intention for a resveratrol food supplement. Furthermore, in the United States the less educated are more likely to be interested. The inverse supplement hypothesis was not confirmed for resveratrol supplements, and even appeared to be indirectly contradicted, given that a food-indulgence orientation was linked to interest in resveratrol.

As an implication for marketing resveratrol supplements, we suggest that apart from clearly communicating the health benefits, messages should target consumers who use CAM and are interested in food as a culinary pleasure. More importantly, from a public-policy perspective, the results suggest that it could be consumers who might have or will develop health problems who are especially drawn to resveratrol supplements—at least when assuming that interest in the enjoyment of food is related to unhealthy eating. Therefore, further research ought to explore whether consumers misperceive resveratrol supplements as a magic solution, with use of the supplement ‘backfiring’, in the sense that they engage in even more unhealthy behaviour after adopting the product [44]. Given that the other factor linked to positive attitudes and adoption intention of resveratrol supplements was CAM usage, this relation might inform how the prospective consumers that are open to resveratrol usage can be approached, which is linked to communication on and about CAM. Further research might also illuminate whether and why those with lower education in the United States might be more drawn to resveratrol. Given that consumers are more and more interested in supplements and that the availability of established, as well as new, variants is increasing, the opportunities and challenges that supplements offer or pose to public health should be monitored by continuous research on market and consumer behaviour.

## Endnote

^a^The ethical guideline document of MAPP Centre for Research on Customer Relations in the Food Sector can be provided on request.

## Additional file

Additional file 1: Table S1. Independent measures not listed in Table 2.

## References

[1] Greger JL (2001). Supplement use is associated with health status and health-related behaviors in the 1946 British birth cohort. Nutr Epidemiol.

[2] Rietjens IMCM, Slob W, Galli C, Silano V (2008). Risk assessment of botanicals and botanical preparations intended for use in food and food supplements: emerging issues. Toxicol Lett.

[3] Kaur S, Das M (2011). Functional foods: an overview. Food Sci Biotechnol.

[4] Sareela M (2000). Functional Foods. Concept to Product.

[5] Howlett J (2008). Functional Foods: From Science to Health and Claims.

[6] EU: Directive on Food Supplements. 2002/46/EC.

[7] Egan B, Hodkins C, Shephard R, Timotijevic L, Raats M (2011). An overview of consumer attitudes and beliefs about plant food supplements. Food Funct.

[8] Beitz R, Mensink GBM, Hintzpeter B, Fischer B, Erbersdobler HF (2004). Do users of dietary supplements differ from nonusers in their food consumption?. Nutr Epidemiol.

[9] Conner M, Kirk SFL, Cade JE, Barret JH (2001). Why do women use dietary supplements? The use of the theory of planned behaviour to explore beliefs about their use. Soc Sci Med.

[10] Furnham AA (2007). Are modern health worries, personality and attitudes to science associated with the use of complementary and alternative medicine?. Br J Health Psychol.

[11] Bishop FL, Lewith GT (2008). Who uses CAM? A narrative review of demographic characteristics and health factors associated with CAM use. Evid Based Complement Alternat Med.

[12] McDonald DD, Nicholson NR (2006). Dietary supplement information and intention to continue and recommend supplements. Int J Nurs Stud.

[13] de Jong N, Ocké MC, Branderhorst HAC, Friele R (2003). Demographic and lifestyle characteristics of functional food consumers and dietary supplement users. Br J Nutr.

[14] Ritchie MR (2007). Use of herbal supplements and nutritional supplements in the UK: what do we know about their pattern of usage?. Proc Nutr Soc.

[15] Rozin P, Spranca M, Krieger Z, Neuhaus R, Surillo D, Swerdlin A (2004). Preference for natural: instrumental and ideational/moral motivations, and the contrast between foods and medicines. Appetite.

[16] Grunert KG (2006). Future trends and consumer lifestyles with regard to meat consumption. Meat Sci.

[17] VitaminShoppe [http://www.vitaminshoppe.com/store/en/vitamins_minerals/index.jsp].

[18] Baur JA, Sinclair DA (2006). Therapeutic potential of resveratrol: the in vivo evidence. Nat Rev Drug Discov.

[19] Chachay VS, Kirkpatrick CMJ, Hickman IJ, Ferguson M, Prins JB, Martin JH (2011). Resveratrol - pills to replace a healthy diet?. Br J Clin Pharmacol.

[20] Szkudelska K, Szkudelski T (2010). Resveratrol, obesity and diabetes. Eur J Pharmacol.

[21] Science (2011). Aging genes: the Sirtuin story unravels. Science news focus. Eur J Pharmacol.

[22] Kopp P (1998). Resveratrol, a phytoestrogen found in red wine. A possible explanation for the conundrum of the ‘French paradox’?. Eur J Endocrinol.

[23] Renaud S, de Lorgeril M (1992). Wine, alcohol, platelets, and the French paradox for coronary heart disease. Lancet.

[24] Rumawas M E, Dwyer JT, Mckeown NM, Meigs JB, Rogers G, Jacques PF. The development of the Mediterranean-style dietary pattern score and its application to the American diet in the Framingham offspring cohort. J Nut Nutr Epidemiol. 2009. doi:10.3945/jn.108.103424. 10.3945/jn.109.103424 PMC2682986 19357215

[25] Timbo BB, Ross MP, McCarthy PV, Lin CT, Lin CT (2006). Dietary supplements in a national survey: prevalence of use and reports of adverse events. J Am Diet Assoc.

[26] Van der Horst K, Siegrist M (2011). Vitamin and mineral supplement users. Do they have healthy or unhealthy dietary behaviours?. Appetite.

[27] Pillitteri JL, Shiffman S, Rohay JM, Harkins AM, Burton SL, Wadden TA (2008). Use of dietary supplements for weight loss in the United States: results of a national survey. Obesity.

[28] Cox DN, Koster A, Russell CG (2004). Predicting intentions to consume functional foods and supplement to offset memory loss using an adaptation of protection motivation theory. Appetite.

[29] Devcich DA, Pedersen IK, Petrie KJ (2007). You eat what you are: modern health worries and the acceptance of natural and synthetic additives in functional foods. Appetite.

[30] Dickson-Spillmann M, Siegrist M, Keller C (2011). Attitudes toward chemicals are associated with preference for natural food. Food Qual Preference.

[31] Barreiro-Hurlé J, Colombo S, Cantos-Villar E (2008). Is there a market for functional wines? Consumer preferences and willingness to pay for resveratrol-enriched red wine. Food Qual Preference.

[32] Lockshin L, Corsi AM (2012). Consumer behaviour for wine 2.0. A review since 2003 and future directions. Wine Econ Pol.

[33] Yoo YJ, Saliba Anthony J, MacDonald JB, Prenzler PD, Ryan D (2013). A cross-cultural study of wine consumers with respect to health benefits of wine. Food Qual Preference.

[34] PlantLIBRA. PLANT food supplements: Levels of intake, benefit and risk assessment. [http://www.plantlibra.eu/web/node/44]

[35] Greenwood JLJ, Murtaugh MA, Omura EM, Alder SC, Stanford JB (2008). Creating a clinical screening questionnaire for eating behaviors associated with overweight and obesity. J Am Board Fam Med.

[36] Hyland ME, Lewith GT, Westoby C (2003). Developing a measure of attitudes: the holistic complementary and alternative medicine questionnaire. Complement Ther Med.

[37] Roininen K, Lähteenmäki L, Tuorila H (1999). Quantification of consumer attitudes to health and hedonic characteristics of foods. Appetite.

[38] Urala N, Lähteenmäki L (2004). Attitudes behind consumers’ willingness to use functional foods. Food Qual Preference.

[39] Chrysochou P, Askegaard S, Grunert KG, Kristensen DB (2010). Social discourses of healthy eating. A market segmentation approach. Appetite.

[40] Kristensen DB, Askegaard S, Jeppesen LH, Anker TB (2010). Promoting health, producing moralisms?. Adv Consum Res.

[41] Grunert KG, Bredahl L, Scholderer J (2003). Four questions on European consumers attitudes toward the use of genetic modification in food production. Innovative Food Sci Emerg Technol.

[42] Rozin P, Fischler C, Imada S, Sarubin A, Wrzesniewski A (1999). Attitudes to food and the role of food in life in the U.S.A., Japan, Flemish Belgium and France: possible implications for the diet-health debate. Appetite.

[43] Raghunathan R, Walker Naylor R, Hoyer WD (2006). The unhealthy = tasty intuition and its effects on taste inferences, enjoyment, and choice of food products. J Mark Manage.

[44] Bolton LE, Americus R, Volpp KG, Armstrong K (2008). How does drug and supplement marketing affect a healthy lifestyle?. J Consum Res.

