# Supplementary material for: Resveratrol food supplements: a survey on the role of individual consumer characteristics in predicting the attitudes and adoption intentions of US American and Danish respondents
Source: BMC Public Health. 2015 Feb 10;15:110. doi: 10.1186/s12889-015-1348-7 (PMC4335419; doi:10.1186/s12889-015-1348-7)
Supplement: Additional file 1: Table S1. — Independent measures not listed in Table 2. [file 12889_2015_1348_MOESM1_ESM.docx]

**Additional file 1: Table S1. Independent measures not listed in table 2**

| **Measure** | **Items** |
| --- | --- |
| Health behaviour | 14 items from [35]:  How many times did you eat fast food (for example, McDonald’s, Burger King, etc.) or at a restaurant yesterday?  How many times did you drink juice yesterday (for example, orange juice, apple juice, fruit smoothie, etc.)?  How many servings (e.g. can, small bottle, glass) of non-diet soda pop did you drink yesterday (for example, Coke, Pepsi, Sprite)?  How many times did you eat vegetables yesterday (for example, broccoli, spinach, greens, salad, etc)?  How many times did you eat fruit yesterday (for example, an apple, an orange, a hand full of grapes, etc)?  How many times do you typically drink juice in one day (for example, orange juice, apple juice, fruit smoothie, etc.)?  How many servings (e.g. can, small bottle, glass) of non-diet soda pop do you typically drink in one day (for example, Coke, Pepsi, Sprite)?  How many times do you typically eat vegetables in one day (for example, broccoli, spinach, greens, salad, etc)?  How many times do you typically eat fruit in one day (for example, an apple, an orange, a hand full of grapes, etc)?  How many times do you typically eat fast food (for example, Chili’s, McDonalds, Burger King, etc.) or at a restaurant in one week?  How many times do you typically eat breakfast in 1 week (7 days)?  When eating restaurant food, how often out on a scale from 1 to 7 do you eat all of the food served to you?  How many days during the past week have you performed physical activity where your heart beats faster and you are breathing harder than normal for 30 minutes or more (in three 10-minute bouts or one 30-minute bout)?  How many days in a typical week have do you perform activity such as this (see above)? |
| Health expectation | 10 items derived from the literature [18–21]:  Taking resveratrol regularly leads to health benefits for all adults.  Taking resveratrol regularly only leads to health benefits for people with certain health problems.  Taking resveratrol regularly only leads to health benefits for people who do not eat healthily enough.  Regular intake of resveratrol strengthens the cardiovascular system.  Regular intake of resveratrol helps preventing obesity-related diseases.  Regular intake of resveratrol contributes to weight loss.  Regular intake of resveratrol strengthens the immune defense system.  Regular intake of resveratrol reduces oxidation of fatty acids (‘oxidative stress’).  Regular intake of resveratrol reduces the risk of certain cancer types.  Regular intake of resveratrol reduces the risk of dementia. |
| Natural product interest | 6 items from [37]:  I try to eat foods that do not contain additives.  I do not worry about additives in my daily diet.  I do not eat processed foods, because I do not know what they contain.  I eat mostly organically grown fruit and vegetables.  In my opinion, artificially flavored foods are not harmful for my health.  In my opinion, organically grown foods are no better for my health than those grown conventionally. |

*Notes*. All statements were measured on a 7-point scale, except for statement 1-11 from the health behaviour which was measured on a 1-9 scale with a coding from 1 = never to 9 = more than seven times.
